# Correspondence

**Published:** 1874-08

**Authors:** J. N. Cheney

**Affiliations:** Newborn, Ga.


Newborn, Ga., May 28, 1874.

*Messrs. Editors:*

I will address this article to you for examination. If you think it worthy of publication, please send it to press; if not, cast it aside. The only peculiarity about the case, is both head presentation and twin placenta. I called to see Mrs. M. P—— on May 2, instant, and found her in the preparatory stage of labor.

She was greatly troubled with difficulty of breathing. She had not slept any, of any consequence, (in a recumbent posture,) for several weeks; all the rest she got was sitting straight up, with her head on a pillow on another chair. I saw that she was very bilious, and that her digestive organs were in a torpid condition. Her nervous system, as well as her breathing, were very much affected by the portal circulation, and all the abdominal and thoracic viscera being compressed by the gravid uterus.

So soon as I informed myself of her condition, I gave her 10 grs. blue pill, combined with one-half gr. sulph. morphia. She then rested very well for two or three hours. When she awoke, she began to have pretty hard pains. I then made an examination, per vaginum, and found the os. rigid and not inclined to dilate, also her pains seemed to be more of a nervous character than true labor. I then gave another very heavy dose of sulph. morphia. She rested finely from the effect of the anodyne, several hours, and then began to have real labor, and by the assistance of wine ergot, soon gave birth to two large boys, both head presentations. The first was still born, the other was born dead; probably had been dead for a day or two. The most peculiar feature in the case were two very large and distinct placentæ, one attached to the fundus of the uterus, the other to the left side. The placentæ were connected together by a large membraneous substance, 6x8 inches in size. No unusual hemorrhage after delivery. Case progressed favorably.

Each child having a separate placenta, it is my opinion they would not have favored each other any more than brothers born at different confinements.

Very respectfully,

J. N. Cheney, M.D.

